# Perimenopausal contraception in Turkish women: A cross-sectional study

**DOI:** 10.1186/1472-6955-6-1

**Published:** 2007-03-08

**Authors:** Nevin H Şahin, Sema B Kharbouch

**Affiliations:** 1 Istanbul University, Florence Nightingale School of Nursing, Department of Obstetric & Gynaecologic Nursing, Istanbul, Turkey; 2 Istanbul University, Cerrahpasa Faculty of Medicine, Women Health Clinic, Istanbul, Turkey

## Abstract

**Background:**

Epidemiologic research has shown that perimenopausal contraception is an important medical issue, because women during the perimenopause still need effective contraception. The objective of the study was to assess the contraceptive choices of perimenopausal Turkish women.

**Methods:**

This is a descriptive cross-sectional study that in a non – random fashion recruited 202 perimenopausal and naturally menopausal women who lived in a suburban area of Istanbul. Women who took part were aged between 45–59 years old. Chief database used to identify the suitable participants in the district. Subjects who voluntarily participated in the study were interviewed in their homes by the researcher. The analysis of the data was evaluated using percentages.

**Results:**

The percentage of sexually active women among the participants was 87.6%. A large majority – 80.2% – of the participants did not have any idea of when they should bring contraception to an end. The method most commonly used was withdrawal (Coitus Interruptus), represented by 38.8%. In regard to the participants' choices of medical contraception, those being utilized were the IUD (24.3%), tubal sterilization (8.9%), condom (5.9%) and COC (6.4%). Additionally, 18% of women used other traditional methods including vaginal lavage, vaginal aspirin, and even inserting a small sponge presoaked with fresh lemon juice or cola deep into the vagina. Among the perimenopausal women who participated, the IUD was the most popular and appropriate contraceptive method.

**Conclusion:**

Most perimenopausal Turkish women are still using traditional methods and women's knowledge about contraception in the menopausal stages is very limited. Health professionals should provide information about perimenopausal contraception.

## Background

Perimenopause marks the transition from normal ovulation to anovulation and ultimately to permanent loss of ovarian function. Fecundity, the average monthly probability of conception, declines by half as early as the mid-forties; however, women during the perimenopause still need effective contraception. Epidemiologic research has shown that perimenopausal contraception is an important medical issue [1-7]. According to the Turkey Demography and Health Survey (TDHS) data, 23.6% and 50.1% of Turkish women between ages 40–44 and 45–49 respectively are not protected against conception [8].

Women in their forties are still potentially fertile, and pregnancy in this age group is attended by increased risks of maternal mortality, spontaneous abortion, fetal abnormalities and perinatal mortality. The maternal mortality rate in women in their forties is four times higher than that among women in their twenties and the rates of spontaneous abortion are also doubled in the forties group [3,5,7,9,10].

There is no research on contraceptive preferences of perimenopausal women in Turkey. The objective of the study was to assess the contraceptive preferences of perimenopausal Turkish women.

## Methods

This study was planned as a descriptive cross-sectional research. The population of this cross-sectional study consisted of women aged 45–59 years who lived in a suburban area of Istanbul. The inclusion criterion for the research sample was the history of amenorrhea for less than 2 years among perimenopausal women aged 45–59 years. Chief database used to identify the suitable participants in the district. During the research period between May and June 2005, 240 women who met the inclusion criteria in the district were invited to the study and 202 women who volunteered to participate in the study were included in the convenience sample non-randomly. Subjects were interviewed in their homes by the researcher.

Turkish women's menopausal ages were taken into consideration when this age group was chosen. Women who had had a surgical menopause were excluded. Women were informed about the aim of the study and information about anonymity, confidentiality, and consent was included in the explanation. The written ethical approval was obtained from the ethical review board of the Istanbul University.

For gathering the data of the study a "Participant Information Form (PIF)" was used. Demographic, gyneco-obstetric and contraception characteristics were collected by the PIF developed by researchers in a 16 – question format. The PIF was administered by the author in face-to-face interviews with each participant in their home.

The analysis of the data obtained from the study was carried out with SPSS for Windows (Statistical Package for Social Science for Windows Version 10.0). Data of the study were analyzed using percentage tests.

## Results

Two hundred and two women completed the study, aged between 45 and 59 years with an overall mean age of 51.23 ± 4.17(X¯
 MathType@MTEF@5@5@+=feaafiart1ev1aaatCvAUfKttLearuWrP9MDH5MBPbIqV92AaeXatLxBI9gBaebbnrfifHhDYfgasaacH8akY=wiFfYdH8Gipec8Eeeu0xXdbba9frFj0=OqFfea0dXdd9vqai=hGuQ8kuc9pgc9s8qqaq=dirpe0xb9q8qiLsFr0=vr0=vr0dc8meaabaqaciaacaGaaeqabaqabeGadaaakeaaieqacuWFybawgaqeaaaa@2E03@ ± SD) years for group as a whole. The participants were predominantly composed of married women (87.6%), with primary school (8 years' education) qualifications (85.4%) from a middle-income group. The majority of them (89.7%) were housewives. The characteristics of the participants are shown in Table 1. Three percent of the participants were nulliparous, 50% had had 1–3 pregnancies, and 47% had had more than 3 pregnancies. The majority of participants suffered from chronic illness such as diabetes (12.4%), cardiovascular (34.7%), endocrine (5.4%), urological (1.5%), respiratory (6.9%), or musculoskeletal (10.9%) disease.

**Table 1:** Characteristics of Participants

Characteristics	**n**	**percent**
**Sexuality**		
Active	177	87.6
No partner	25	12.4
**Chronic Disease**		
Diabetes	25	12.4
Cardiovascular Disease (hypertension)	79	39.1
Other (endocrine, urologic, respiratory, muscular-skeleton)	62	30.7
Healthy (No Chronic Disease)	36	17.8
**Routine gynecological control**	82	40.6
**Regular exercise **(3 times/per week/30–60 minute)	39	19.3
**Smoking **≥ 20(daily)	43	21.3
**Alcohol consumption: **Rarely (Social drinker)	16	8.4
	**Mean**	**SD**
Age (year)	51.2	4.1
Body Mass Index	28.4	4.3
Number of Births	2.6	1.4
Number of Abortions	0.4	0.8

Information concerning the participants' awareness of the correct timing of giving up contraception is presented in Table 2. The majority of the participants did not have any clear idea about the appropriate time for abandoning contraception.

**Table 2:** When should contraceptive methods be given up after menopause?

Contraceptive Termination Time	n	Percent
I don't know	162	80.2
One year later	36	17.9
Two years later	4	1.9
Total	202	100

Regarding the most recent contraception methods used by the perimenopausal participants, the withdrawal method was most often used (38.8%). Other traditional methods were being used in a widespread manner (18%), including vaginal lavage, vaginal Aspirin^® ^(salisilik asit tab.), and even the insertion of a small sponge soaked in with fresh lemon juice or cola deep into the vagina. As far as medical methods are concerned, the methods commonly used were IUD (24.3%), tubal sterilization (8.9%), condom (5.9%) and COC's (6.4%).

## Discussion

Perimenopause is defined as a transition from childbearing and the requirement for contraception to the infertility of menopause. According to the results of the study, most of the perimenopausal participants were sexually active (87.6%). The majority of participants had a chronic disease such as diabetes, cardiovascular (hypertension), endocrine, urological, respiratory, or musculo-skeletal diseases. The degree of parity and other characteristics of the participants were similar to the same age group in the TDHS data. Generally, women living in Turkey do not attend a gynecological clinic regularly for check ups [8]. They need counseling related to health promotion and the regulation of fertility.

Women in their forties are still potentially fertile, and pregnancy in this age group is attended by increased risks of maternal mortality, spontaneous abortion, fetal anomalies and perinatal mortality. In developing countries, these risks are compounded by high number of pregnancies and poor medical care. A recent survey reported that in the United States, 41% of pregnancies in women between ages 35 and 39 years and 51% of pregnancies in women at ages 40 years and older are unintended [2,3,5,10,11]. According to the TDHS data, 12% of pregnancies in women occur between ages 40 and 44 and 2% of pregnancies in women occur between ages 45 and 49 in Turkey. Most of these pregnancies are unintended [8]. Four fifths of the participants did not have any idea of when they should bring contraception to an end. Most clinicians advise women to continue using contraception for 12 months after menstruation has ceased. Some recommend that women less than 45 years old continue contraception for 2 years after menstruation has ceased. In the literature, it is reported that women can stop taking non-hormonal methods after 6 months of amenorrhea if women experience hot flushes. In the absence of classical menopausal symptoms, however, they should wait for 12 months prior to stopping contraception use. Women should still use contraception until the menopause [6,7,9]. Unfortunately, the majority of the participants did not have any clear idea about the appropriate time for abandoning contraception, so the risk of unintended pregnancies was high. Health professionals must use every opportunity to give the necessary information to perimenopausal women.

The method selected primarily was the traditional withdrawal (38.8%) and other traditional methods. Among the perimenopausal women, the IUD (24.3%) was the most popular medical method. Other medical methods being utilized were tubal sterilization (8.9%), condom (5.9%) and COC (6.4%) (Table 2). Contraception for women in this age group has special risks and benefits: both should be balanced in order to choose between the different options available.

Withdrawal which is considered to be a very common contraceptive method nationally, is practiced at rates of 29.5% among women between ages 40 – 44 and 20.9% among women between ages 45 – 49 [6,8].

Most perimenopausal Turkish women are still using traditional methods like a vaginal lavage, vaginal Aspirin^® ^(acetylsalicylic acid tab.), and sponge soaked in fresh lemon juice or cola deep into the vagina. Health professionals should provide knowledge of perimenopausal contraception, including its noncontraceptive effects and health risks during fertility regulation counseling.

IUD usage rates in Turkey are 19.2% and 11.1% among women between ages 40 – 45 and 45 – 49 respectively [8], being the most commonly used medical method among all age groups. According to the WHO's Medical Eligibility Criteria for Contraceptive Use [12] studies done on the IUD -which does not have any contraindications related to age- among users over 35 years old, showed that incidences of side affects of the device on this age group were low. In our study, IUD usage rate was higher than TDHS (24.3%). The participants who live in Istanbul can reach services and medical contraceptive methods more easily than others who live in other areas[8].

At the 1994 WHO Scientific Group's menopause assembly in Geneva, it was decided that the levonorgesterol intrauterine system was a very suitable contraceptive choice for perimenopausal women because it is associated with minimal bleeding problems [7]. It is not a popular contraceptive method in Turkey. It is an expensive alternative only available to attendants of private clinics [8].

According to the TDHS data, the usage rates of COC's are 3.3% among women aged 40–44 years and 1.3% among women between ages 45–49 in Turkish women [8]. In this study the usage rates of COC were shown to be higher than the total of other current medical methods in Turkey (6.4%) such as the IUD. This positive situation may be related to the fact that the participants who live in Istanbul, a big city, have access to the services and medical contraceptive methods more easily than women in other areas.

Recent epidemiological and clinical pharmacological studies have indicated the safety of extending the use of combined oral contraceptives (COCs) beyond the age of 35 years and until menopause. Decisions regarding oral contraceptive choice should depend on many factors, including health risks and non-contraceptive benefits. Clinicians should pay attention to overweight based on BMI, cigarette smoking and chronic diseases in Turkish perimenopausal women. Cardiovascular diseases, especially diabetes and hypertensive diseases were common in the participant women. A woman needs to consider her age, health status, menstrual history, and sexuality, risk of sexually transmitted diseases (STDs), past contraception use, lifestyle, obstetric history, and attitude toward abortion when choosing a contraceptive method during the perimenopausal years. Clinicians should educate women in this age group about the benefits of oral contraceptives while addressing misperceptions to allow more women into this therapeutic option. Numerous benefits unrelated to contraception may be attractive to women of this age group [1,3,4,6,7,9,11,13].

Women who have reasons for avoiding COCs can use progestogen-only contraceptives like pills, depot injectables and implants. Both copper-releasing and levonorgestrel-releasing IUDs (LNG-IUD) combine the advantages of high efficacy and long term effects [12]. Among perimenopausal women, it is only the progestin containing methods that are the least popular in Turkey (0.1%). The pharmacies in Turkey do not currently keep the Mini Pill. Besides, perimenopausal women in Turkey show little interest at present in the recently introduced method, depot enjectables [8].

Besides being a contraceptive, the condom has the added benefit of protection against STDs. Women who are between ages 35–39 and using the barrier method have a 1.1% risk of becoming pregnant [7]. In Turkey, the rates of condom usage among women who are between ages 40–44 and 45–49 are 10.0% and 5.7% respectively [8]. Similarly, the rate of condom usage among perimenopausal women in the study was 5.9%.

The female condom can be a source of irritation in older age groups who may perceive themselves as unattractive from the esthetic point of view [7]. None of the participants used the female condom, which will require special promotional campaigns in order to become a popular method in Turkey.

According to the TDHS data, usage rates for the diaphragm in Turkey are 10% among women at 40 – 44 years old and 0.9% among women at 45 – 49 years old [8]. The rate of diaphragm usage in this study was very low. However, the diaphragm could be an appropriate alternative method with the advantage of having no systemic side effects for those perimenopausal women with chronic diseases. Health professionals should promote knowledge of medical methods for contraception during fertile years.

In the United States and Australia in particular, male or female sterilization is the most popular method of contraception for women over the age of 35. Failure rates are between 0.75% and 3.65%. In the United Kingdom, more than 40% of couples over the age of 40 choose sterilization as a method of family planning. This is true for other developed countries as well [[3,9],14]. According to the TDHS data in Turkey, the female sterilization rate is 10.9% among women between ages 40 to 44 and 6.5% among women between 45 and 49 [8]. The equivalent rate in the study was 8.9%. Although male or female sterilization has been legal since 1983, sterilization is not very common in the Turkish population. This approach is not viewed as a culturally acceptable method [8]. In this study the rates of female sterilization were shown to be higher than that currently in the rest of Turkey as a whole but they were lower than in other countries.

Hormone Replacement Therapy (HRT) alone is not a form of contraception, as the dosage of estrogen is not great enough to stop ovulation from occurring. If contraception is still required, a woman may safely continue taking oral contraceptives until 55 years old, when conception is extremely unlikely [1,7,11]. HRT use is not widespread in Turkey [15]

## Conclusion

Although many perimenopausal contraception choices are available in Turkey, the rates of contraception use are very low. As a result, unintended pregnancy rates are high. Finally, contraceptive choice should be carefully guided by considering personal and family health history, individual preferences and previous experience. Health professionals should promote knowledge of all medical methods for fertility regulation during fertile years.

## List of abbreviations

BMI: Body Mass Index

CI: Coitus Interruptus

COC: Combined Oral Contraception

CVD: Cardiovascular Disease

TDHS: Turkey Demography and Health Survey (2004)

HRT: Hormone Replacement Therapy

IUD: Intrauterine Device

PID: Pelvic Inflammatory Disease

STD's: Sexually Transmitted Diseases

WHO: World Health Organization

## Competing interests

The author(s) declare that they have no competing interests.

## Authors' contributions

NHS and SBK made substantial contributions to conception and design

SBK conducted the acquisition, analysis and interpretation of data

NHS was involved in drafting the manuscript and revising it critically for important intellectual content

NHS and SBK have given final approval of the version to be published.

## Pre-publication history

The pre-publication history for this paper can be accessed here:


